# Awareness and practice of breast self-examination among female staff at Babcock University, Nigeria

**DOI:** 10.3332/ecancer.2023.1615

**Published:** 2023-10-30

**Authors:** Julius Olatade Maitanmi, Olaide Fadare, Moyosola Kolawole, Damilare Matthew Aduroja, Damilola M Faleti, Bukola Titilope Maitanmi, Oluwadamilare Akingbade

**Affiliations:** 1 School of Nursing, Babcock University, Ilishan-Remo, Ogun State 121103, Nigeria; 2 Institute of Nursing Research, Osogbo 230262, Osun State, Nigeria; 3 The Nethersole School of Nursing, Faculty of Medicine, The Chinese University of Hong Kong, Hong Kong 999077, China

**Keywords:** awareness, breast self-examination (BSE), practice, female

## Abstract

**Background:**

Despite the ever-growing breast cancer awareness campaigns in Nigeria, the practice of breast self-examination (BSE) continues to vary widely among women. We aimed to assess breast cancer awareness and practice of BSE among female staff at Babcock University, Ogun State, Nigeria.

**Methods:**

The cross-sectional descriptive design was adopted for this study. Data were collected using a structured questionnaire administered to 160 respondents. Data analysis was done using Statistical Package for Social Sciences version 23.

**Results:**

Although the majority of the respondents were highly aware of breast cancer (78.12%) and had good knowledge about BSE (96.9%), their practice of BSE was low. Only 11.3% always examine their breasts in the mirror by raising their hands over their head, looking at their breasts and examining it in a circular motion; 56% rarely looked for puckering, colour changes and dimpling of the skin when examining their breasts in the mirror, although 53.8% sometimes squeezed their nipples and looked for discharge when they examined their breasts.

**Conclusion:**

Knowledge and positive opinions toward preventive strategies for breast cancer should not be interpreted as readiness for practice. Educational intervention programs emphasising the importance of early detection in managing breast cancer should be reinforced to birth the desirable change.

## Introduction

Breast cancer continues to be the primary cause of mortality among women, following lung cancer, due to its significant impact on morbidity and mortality rates [1]. Breast cancer is the most often diagnosed cancer worldwide and is the primary cause of mortality among women. It constitutes around 23% of all cancer cases and 14% of cancer-related deaths. The yearly incidence of breast cancer is estimated at 3%, while the mortality rate stands at 1.8%. This phenomenon represents a significant global public health concern, affecting approximately 2.1 million women annually [2, 3]. According to estimates made in 2018, the number of fatalities resulting from breast cancer among women was approximately 627,000. The incidence of breast cancer among women in Nigeria has exhibited a consistent upward trend, with the number of individuals at risk rising from an estimated 24.5 million in 1900 to roughly 40 million in 2010. Projections indicate that this figure is expected to surpass 50 million by 2020 [4]. Given these circumstances, it is anticipated that around one out of every 26 women will receive a breast cancer diagnosis during their lifetime, with the majority of cases manifesting in women in their premenopausal stage. The prevalence of breast cancer in Nigeria has experienced a substantial increase, reaching a rate of 54.3 per 100,000 individuals. This represents a 100% jump over the past decade [5].

Furthermore, it has been observed that Western European and North American regions exhibit a cancer survival rate of 70% among individuals aged 30–40 years. This rate significantly surpasses Nigeria's, where the chances of breast cancer survival are only 10%. Consequently, this disparity in survival rates negatively impacts the life expectancy of the at-risk population in Nigeria [6]. The prevalence and mortality rate of breast cancer and the substantial financial burden of treatment necessitate that health authorities and policymakers prioritise providing comprehensive access to all available treatment modalities. This approach is crucial to enhance the likelihood of improved survival rates [4]. The lack of knowledge exacerbates the situation and impedes the capacity to effectively address the escalating prevalence, notwithstanding Nigeria's heightened rate of breast cancer awareness campaigns. The association between delayed hospital presentation of breast cancer patients and their poor inclination to seek medical care has been observed in a study [4, 7]. It is well acknowledged that early detection of a disease condition has a notable impact on the prognosis and mitigates the progression of complications and disability associated with this ailment. The early detection of breast cancer has been associated with a decrease in both the incidence of illness and death rates and a reduction in the financial burden of treatment. Various techniques have been utilised in the past and continue to be utilised for the timely identification of breast cancer. These techniques encompass breast self-examination (BSE), clinical breast examination (CBE) and mammography screening [8, 9].

BSE is a type of physical assessment done by women that enables them to know and feel the texture of their breasts as they check for lumps, shape, size and contour. Furthermore, this is done so that the woman can learn the topography of her breasts, know how her normal breasts feel and detect changes in them if any occur in the future. Studies have shown that BSE positively affects detecting breast cancer early [9]. BSE is best employed in areas where CBE and mammography cannot be achieved. Even though it is not scientifically proven to be the best method in the early detection of breast cancer among the female population, it is best recommended in low-resource areas because it is free, private, painless, easy, safe and does not involve using any equipment. Also, the American Cancer Society recommends that women from the age of 20 years onward should be educated on the importance of performing BSE every month [10].

Studies have shown that improved awareness, like the improved media announcement about monthly BSE and risks of breast cancer, has significantly improved the early detection associated with breast cancer, leading to better health management [11, 12]. Therefore, there is a need to discover how many more women are yet to understand what breast cancer is and how to practice the early detection method, that is, BSE.

Although BSE is readily affordable and more readily available than any other method of early detection of breast cancer in our environment, there is a paucity of evidence regarding the awareness of female University staff on BSE and its practice, which prompted this study which will investigate breast cancer awareness and practice of BSE among female staff at Babcock University. This study will provide evidence to guide future intervention programs on breast cancer and BSE in the community.

## Methods

This study used a quantitative approach with a cross-sectional descriptive design to determine breast cancer awareness and practice of BSE among female staff in Babcock University, Ilishan-Remo, Ogun State, a southwestern part of Nigeria. The target population consists of non-teaching female staff who work at Babcock University. The study used a purposive sampling technique to select the respondents from different units until the sample size was reached. A sample size of 164 was used, which was determined using the Taro Yamane formula.

Using Taro Yamane formula (*n* = *N*/1 + *N*(*e*^2))

*N* = 220

*e* = 0.05

*n* = ?

*n* = 220

1 + 220 (0.05^2)

*n* = 220

1 + 220 (0.0025)

*n* = 220

1.55

*n* = 141.93, approximated to 142

*n* = 142

10% Attrition rate = *N*/100 * 10

= 220 * 10

100

= 2.2 * 10

= 22

Sample size = *n* + 22 = 142 + 22

Sample size = 164.

A total of 164 questionnaires were distributed during official work hours for 1 month (October–November 2020); however, only 160 could be retrieved, giving a response rate of 97.6%. The data was collected using a semi-structured self-administered questionnaire adapted into four sections. Section A of the questionnaire was designed to elicit information on the demographic characteristics of the respondents. Section B focused on the awareness of female staff on breast cancer, while section C focused on the knowledge of the female staff on BSE. Section D measured the practice of BSE among the female staff. The validity and reliability tests of the instrument were ensured. A pre-test was carried out on a small population to achieve the instrument's reliability; 16 copies of the instrument were pre-tested on a different set of female staff. The reliability test was ascertained using Cronbach‘s alpha method, and a result of −0.833 was obtained. The 16 participants used in the pre-test were not included in the final study. The questionnaire was written in English, which made interaction with the participants easier and enabled the participants to give accurate information.

### Data analysis

Data collected were analysed using the Statistical Package for Social Sciences version 23. The data was presented, organised and summarised numerically using descriptive statistics of frequencies and percentages.

### Ethical consideration

A proposal was submitted to the Babcock University Health Research Ethics Committee. Participants were adequately informed about the study's purpose and the questionnaire's content. There was no overt or covert coercion, as participants were allowed to decide whether to participate in the study. Guidelines for the completion of the questionnaire were explained to the participants. Privacy and respect for human dignity were followed during and after the data collection procedure. Respondents were assured of confidentiality and anonymity.

## Results

Table 1 reveals that nearly half of the respondents (46.3%) are between 26 and 35 years old. Also, more than half of the respondents (53.8%) are Christians. Additionally, an appreciable number of the respondents (57.1%) are from the Yoruba ethnic group, and 53.8% are married. This is plausible as the location of the study is the southwestern region of Nigeria, which Christians and Yorubas popularly dominate. Also, 65% of the respondents have attained a tertiary level of education.

Results from Table 2 show that the majority of the respondents (88.8%) have heard about breast cancer; also (42.5%) of the respondents confirmed that they heard from the media, while (35.0%) heard from friends. Furthermore, all (100%) respondents demonstrated that they had been health-educated on breast cancer. Most respondents (65%) said breast cancer cannot develop only in one breast. Similarly, 42.5% of the respondents said women younger than 30 cannot get breast cancer. Also, the majority (88.8%) said breast cancer is more common in women with big breasts. Also, 57.5% of the respondents agreed that a woman’s chance of surviving breast cancer is not very low, even if it is noticed early. Also, 57.5% of the respondents opined that breast cancer is not hereditary, while 46.3% indicated that injury to the breast cannot lead to breast cancer.

The awareness score was classified into three categories in which 9.38% have low awareness, 12.5% have average level of awareness and 78.12% have high level of awareness about breast cancer, as illustrated in Table 3.

From Table 4, the knowledge score was classified into three categories in which 96.9% have a high level of knowledge about BSE while 3.1% have an average level of knowledge. None had a low level of knowledge. This is logical since the majority of the respondents have achieved tertiary education.

Table 5 shows that 66.3% of the respondents confirmed that they never examined their breasts in the mirror by raising their hands over their heads and looking at their breasts. Also, for how often they look for puckering when examining their breasts, 35.0% indicated that they rarely do it. It was indicated that 53.8% sometimes squeeze their nipples and look for discharge, colour changes and dimpling of the skin when examining their breasts. Another 53.8% submitted that they rarely feel their breasts by examining them in a circular motion or a pattern that allows them to cover the entire breast. Slightly more than half of the respondents (35.0%) said they never examine their breasts while standing, while half of the respondents (50%) sometimes examine each breast while lying down with a pillow under the shoulder of the breast being examined.

Table 6 shows the practice rating scale used in measuring good or poor practice of BSE.

The practice score in Table 5 was rated on a 27-point rating scale as shown in Table 6, with a mean score of 16.3. Scores of 17 and above (53.8%) were rated as good practice, and scores below 17 (46.3%) indicated poor practice of BSE. It can therefore be concluded that majority of the respondents exhibited poor practice of BSE.

Table 7 shows that age (*x*^2^ = 105.205^a^, *p* = <0.05), Religion (*x*^2^ = 160.000^a^, *p* < 0.05), Tribe (*x*^2^ = 71.852^a^, *p* < 0.05) and level of education (*x*^2^ = 100.125^a^, *p* = <0.05), were significantly associated with the practice of BSE among female staff in a Nigerian university. From the result, the tribe was significant probably because the Yoruba tribe was more as the study was conducted in Babcock University, which is a Yoruba-dominated higher institution. Furthermore, the majority of the respondents were Christians, and the majority displayed a good self-examination practice. This is not surprising as most participants are Christians working in a Christian institution that upholds healthy practices.

Additionally, it can be deduced from the table that the Yoruba tribe has the largest percentage of participants who often practice BSE; this may have resulted from the fact that this tribe are highly educated compared to other tribes in Nigeria.

## Discussion

This study aimed at investigating the breast cancer awareness, knowledge of BSE and levels of BSE practices among female staff of Babcock University. The study revealed that, the majority of the respondents are highly aware of breast cancer. This finding is consistent with the study that examined women's knowledge, attitudes and practices towards breast cancer in Benin City, Nigeria, where it was discovered that most respondents (90.5%) have a general knowledge of breast cancer [13]. Furthermore, this finding corroborates the findings of a study that evaluated BSE practice among female secondary school students in Osogbo, Western Nigeria, where a good number of the respondents (97.5%) were discovered to have a general knowledge of breast cancer [14]. However, the overall women's knowledge of breast cancer was found to be low in a study carried out on awareness, attitudes and practices of women about breast cancer in Niger State, Nigeria, as only 41.2% of the women were aware of breast cancer. In another study conducted on breast cancer knowledge and practice of BSE among female university students, 44.2% of participants were discovered to have good knowledge of breast cancer [15, 16]. This variance can be because some women are still not yet exposed to adequate information on breast cancer, and this suggests that more awareness programs should be carried out to keep them further informed [17, 18].

Furthermore, this study's findings revealed that most respondents had a high knowledge of BSE. This finding is similar to the findings of the study about knowledge, attitude and practice of BSE among female secondary school teachers in Ilorin, Nigeria, where it was reported that (95.6%) of their study population are aware and knowledgeable about breast cancer self-examination. Furthermore, the study on the knowledge, practice and attitude of breast self, clinical breast and mammographic examinations amongst medical doctors in Bayelsa State also revealed that the respondents (80.9%) have high knowledge of BSE [19, 20]. This high knowledge should be further encouraged and strengthened because one of the most important strategies for achieving early detection of breast cancer is through BSE, which cannot be practised effectively if there is a lack of adequate knowledge about it [19]. However, a study by Ajayi and Faleti [21] reported a lower level of knowledge of market women in Ekiti, Nigeria, regarding BSE, 70% and a 40% level of practice. This suggests that women with a low level of education should be considered when designing interventions to improve knowledge and practice of BSE.

The result of the practice of BSE shows that the majority do not engage in this practice despite the high awareness of breast cancer and high knowledge about BSE. This was also revealed in a study carried out to examine the knowledge, attitude and practice of BSE among women in Rivers State, which found that respondents have heard about BSE but do not practice it [22]. It was also discovered in two studies conducted in South West Nigeria that a good number of those who had good knowledge about BSE were yet to translate this good knowledge into the practice of BSE. This suggests that good knowledge and positive opinions toward preventive strategies should not be interpreted as readiness to practice secondary preventive strategies [14, 21]. There is, however, a solid indication that practice increases as the level of knowledge increases; therefore, in addition to awareness programs, educational interventions that teach the step-by-step practice of BSE are necessary to increase the practice of BSE [14, 23]. It is important to note that educational interventions have also improved cervical cancer screening [24, 25]. These interventions can also consider addressing the lifestyle of women diagnosed with breast cancer [26].

Furthermore, to address the gap in practice, the usage of mobile phones can be explored. Various studies have revealed improved BSE competencies through mobile phones. A study conducted to strategise design for positive BSE experience through mobile phone applications increased women’s positive affect towards BSE while decreasing their negative affect simultaneously [27]. Also, the use of smartphone applications led to a fourfold increase in the practice of BSE among the participants in the intervention group of a study that recommended that women use mobile applications to improve their performance and health beliefs regarding BSE [28]. Similarly, as a high level of mobile phone usage has been reported among this population and Nigerians generally during the COVID-19 pandemic, and Nigerian nurses have successfully designed digital interventions using the internet, mHealth interventions can be designed in Nigeria to teach women BSE techniques [29–32].

## Limitations

Although this study has addressed a research gap by highlighting the awareness and practice of BSE among female staff of a private University in Nigeria, conducting the study only in one private university limits generalisation. This limitation should be considered when interpreting the findings. Also, this warrants more studies in other educational institutions with larger sample sizes to confirm these findings.

## Conclusion

Gaps in BSE discovered in this study should be addressed by the relevant stakeholders. In the study setting, health professionals like nurses and doctors could be contacted to design educational interventions to improve the competencies of the female staff in BSE. Additionally, health professionals should identify other challenges women face in carrying out BSE practices and advise on how to surmount these challenges. Male staff in this institution could also serve as agents to reach out to the larger community by extending information about this subject to their wives and sisters. Similarly, the Ministry of Health could liaise with international organisations to secure grants for non-governmental organisations and civil society organisations to design community-based programmes to educate women on BSE techniques.

## Author contributions

**Conception and design:** Julius Olatade Maitanmi and Olaide Fadare.

**Data analysis and interpretation:** Julius Olatade Maitanmi and Olaide Fadare.

**Manuscript draft:** Moyosola Kolawole, Damilare Matthew Aduroja, Damilola M. Faleti, Bukola Titilope Maitanmi and Oluwadamilare Akingbade.

**Critical revision of the manuscript:** Moyosola Kolawole, Damilare Matthew Aduroja and Oluwadamilare Akingbade.

**Final approval of the manuscript:** Julius Olatade Maitanmi, Olaide Fadare, Moyosola Kolawole, Damilare Matthew Aduroja, Damilola M. Faleti, Bukola Titilope Maitanmi and Oluwadamilare Akingbade.

## Conflicts of interest

The authors have no conflict of interest to declare.

## Funding

The authors received no financial support for the research, authorship, and publication of this article.

## Figures and Tables

**Table 1.** Respondent’s demographic data.

Respondents = 160			
Demographic	Category	Frequency (*f*)	Percentage (%)
Age	16–25	18	11.3
	26–35	74	46.3
	36–45	50	31.3
	45 and above	18	11.3
Religion	Christianity	86	53.8
	Islam	74	46.3
	Traditional	0	0
Tribe	Yoruba	92	57.5
	Igbo	50	31.3
	Hausa	18	11.3
Marital status	Married	86	53.8
	Single	74	46.3
Educational qualification	Secondary	56	35
	Tertiary	104	65

**Table 2.** Frequency distribution showing respondent’s level of awareness of breast cancer.

Category		Respondents 160	
		Frequency F	Percentage (%)
Have you heard about breast cancer?	Yes	142	88.8
	No	18	11.3
	I don’t know	0	0
If yes, what was your source?	Media	68	42.5
	Friend	56	35
	health centre	36	22.5
Have you ever been health educated on breast?	Yes	160	100.0
	No	0	0
	I don’t know	0	0
Breast cancer can only develop in one breast?	No	104	65.0
	I don't know	56	35
Women younger than 30 years do not get breast cancer?	Yes	68	42.5
	No	92	57.5
	I don’t know	0	0
Breast cancer is more common in women with big breast?	Yes	142	88.8
	No	18	11.3
A woman chance of surviving breast cancer is very low even if it is noticed early?	Yes	50	31.3
	No	92	57.5
	I don't know	18	11.3
Breast cancer can be hereditary?	Yes	50	31.3
	No	92	57.5
	I don't know	18	11.3
Injury to the breast can ensure breast cancer?	Yes	68	42.5
	No	74	46.3
	I don't know	18	11.3

**Table 3.** Level of awareness of breast cancer.

Knowledge	Frequency	Percentage
High level of awareness (71–100)	125	78.12
Average level of awareness (41–70)	20	12.5
Low level of awareness (0–40)	15	9.38
Total	160	100

**Table 4.** Level of knowledge of breast self-examination.

Knowledge	Frequency	Percentage
High level of knowledge (71–100)	155	96.9
Average level of knowledge (41–70)	5	3.1
Low level of knowledge (0–40)	0	0
Total	160	100

**Table 5.** Frequency of respondents’ practice of BSE.

Variables		Frequency (*f*)	Percentage (%)
How often do you examine breast in the mirror by raising your hands over your head and looking at your breast?	Never	106	66.3
	Rarely	18	11.3
	Sometimes	18	11.3
	Always	18	11.3
When you examine your breast in the mirror, how often do you look for puckering, colour changes and dimpling of the skin?	Never	18	11.3
	Rarely	56	35
	Sometimes	36	22.5
	Always	50	31.3
When you examine your breast how often do you squeeze your nipples and look for discharge?	Never	18	11.3
	Rarely	56	35
	Sometimes	86	53.8
When you examine your breast how often to you feel your breast by examining in a circular motion or a pattern which allows you to cover the entire breast?	Never Rarely	56 86	35 53.8
	Always	18	11.3
When you examine your breast, how often do you feel the area under your arms to the edge of your breast	Never	56	35
	Rarely	68	42.5
	Sometimes	36	22.5
When you examine your breast how often do you examine your breast while standing	Never	56	35
	Rarely	34	21.3
	Sometimes	52	32.5
	Always	18	11.3
When examining your breast, how often do you examine each breast while lying down with a pillow under the shoulder of the breast being examined?	Never	2	1.3
	Rarely	60	37.5
	Sometimes	80	50
	Always	18	11.3

**Table 6.** Level of BSE practice rating scale.

Level of practice	Frequency	Percentage
*x̅ =* 16.3 ± 5.50	Good practice (0–16)	74 (46.3%)
Min = 0	Poor practice (17–27)	86 (53.8%)
Max = 27	Total	160 (100%)

**Table 7.** Cross tabulation showing the relationship between socio-demographic characteristics of female staff and practice of BSE among female staff in a Nigerian university.

	PRACTICE				
	poor practice	Good practice	*X* ^2^	df	(*p*-value)
Age					
16–25	56 (75.7%)	18 (24.3%)			
26–35	18 (100.0%)	0 (0.0%)	105.205^a^	3	0.000
36–45	0 (0.0%)	50 (100.0%)			
45 and above	0 (0.0%)	18 (100.0%)			
Religion					
Christian	0 (0.0%)	86 (100.0%)	160.000^a^	1	0.000
Muslim	74 (100.0%)	0 (0.0%)			
Tribe					
Yoruba	56 (60.9%)	36 (39.1%)			
Igbo	0 (0.0%)	50 (100.0%)	71.852^a^	2	0.000
Hausa	18 (100.0%)	0 (0.0%)			
Level of education					
Secondary	56 (100.0%)	0 (0.0%)			
Tertiary	18 (17.3%)	86 (82.7%)	100.125^a^	1	0.000

a Significant 0.05 level of significance
