# Awareness and practice of breast self-examination among female staff at Babcock University, Nigeria

**DOI:** 10.3332/ecancer.2024.1652

**Published:** 2024-01-05

**Authors:** Julius Olatade Maitanmi, Olaide Fadare, Moyosola Kolawole, Damilare Matthew Aduroja, Damilola M Faleti, Bukola Titilope Maitanmi, Oluwadamilare Akingbade

**Affiliations:** 1 School of Nursing, Babcock University, Ilishan-Remo, Ogun State 121103, Nigeria; 2 Institute of Nursing Research, Osogbo, Osun State 230262, Nigeria; 3 Faculty of Medicine, The Nethersole School of Nursing, The Chinese University of Hong Kong, Hong Kong 999077, China

**Keywords:** awareness, breast self-examination (BSE), practice, female

The text related to Table 6 was incorrect and should be as follows:

Table 6 shows the practice rating scale used in measuring good or poor practice of BSE.

The practice score in Table 5 was rated on a 27-point rating scale as shown in Table 6, with a mean score of 16.3.

Scores of 17 and above (53.8%) were rated as poor practice, and scores below 17 (46.3%) indicated good practice of breast self-examination.

It can therefore be concluded that the majority of the respondents exhibited poor practice of BSE.

## Figures and Tables

**Table 6.** Level of BSE Practice Rating Scale.

Level of practice	Frequency	Percentage
*x̅* = 16.3 ± 5.50	Good practice (0–16)	74 (46.3%)
Min = 0	Poor practice (17–27)	86 (53.8%)
Max = 27	Total	160 (100%)

